# Mixing it up in the ocean carbon cycle and the removal of refractory dissolved organic carbon

**DOI:** 10.1038/s41598-018-20857-5

**Published:** 2018-02-07

**Authors:** Yuan Shen, Ronald Benner

**Affiliations:** 1 School of the Earth, Ocean and Environment, University of South Carolina, Columbia, South Carolina 29208 USA; 2 Department of Biological Sciences, University of South Carolina, Columbia, South Carolina 29208 USA; 3 Present Address: Ocean Sciences Department, University of California, Santa Cruz, California 95064 USA

## Abstract

A large quantity of reduced carbon is sequestered in the ocean as refractory dissolved molecules that persist through several circuits of global overturning circulation. Key aspects of the cycling of refractory dissolved organic carbon (DOC) remain unknown, making it challenging to predict how this large carbon reservoir will respond to climate change. Herein we investigate mechanisms that remove refractory DOC using bioassay experiments with DOC isolated from surface, mesopelagic and deep waters of the Atlantic Ocean. The isolated DOC was refractory to degradation by native microbial communities, even at elevated concentrations. However, when the refractory DOC was introduced to a series of novel environmental conditions, including addition of a labile substrate, a microbial community from coastal waters and exposure to solar radiation, a substantial fraction (7–13%) was removed within 1.5 years. Our results suggest that while refractory molecules can persist in the ocean for millennia, removal is rapid when they encounter their fate. The observed and projected climate-induced slowdown of global overturning circulation could reduce the exposure of refractory molecules to disparate removal processes. Assuming a constant rate of production, the reservoir size of refractory DOC could increase as overturning circulation slows, providing a negative feedback to rising atmospheric CO_2_.

## Introduction

A vast reservoir (~660 Pg) of dissolved organic carbon (DOC) resides in the ocean, and the cycling of this carbon remains an enigma after decades of inquiry^1–3^. Investigations of the chemical nature of DOC reveal an incredibly diverse and complex mixture of predominantly small molecules (<1 kDa) with an average radiocarbon age of about five thousand years^3–5^. This long-lived DOC reservoir sequesters carbon dioxide from the atmosphere and likely plays an important role in shaping global climate, but little is known about the stability of the reservoir. Seawater bioassay experiments provide insight about the microbial utilization of DOC, and overall they indicate most marine DOC is very resistant to degradation and is considered to be refractory^1,6,7^. The long-term persistence of DOC in the ocean is widely recognized, but the mechanisms responsible for its removal and the processes regulating the size of this large carbon reservoir are not well understood^8^.

The persistence of marine DOC has been related to environmental conditions and microbial communities^9,10^, the intrinsic composition and structure of molecules^11,12^ and low concentrations of molecules that are below a threshold for microbial utilization^13,14^. Diverse and versatile microorganisms distributed throughout the ocean are the primary consumers of DOC, and it appears abiotic processes also play an important role in the removal of refractory molecules. Photochemical processes degrade and alter molecular structures of DOC in ocean surface waters^15^, and elevated temperatures in hydrothermal vent systems pyrolyze DOC in deep waters^16,17^. Thus, the removal of DOC from the ocean appears to occur through disparate processes that are heterogeneously distributed in space and time and connected by physical transport processes.

Building on insights from prior studies, we examined the removal of refractory DOC under varying environmental conditions using experimental approaches. Solid phase extraction (C-18) was used to isolate refractory molecules from surface (50 m), mesopelagic (500 m), and deep (1200 m) waters of the Atlantic Ocean. Bioassay experiments with concentrated C-18 extracts were used to verify the refractory nature of the isolated DOC, followed by a series of manipulations to investigate the removal of refractory DOC under different scenarios, including substrate priming and exposure to irradiation and distinct microbial communities (Supplementary Fig. 1). The sequential exposure of refractory DOC to different removal processes resulted in substantial removal of refractory DOC and provides novel insights about the regulation of the ocean carbon cycle.

## Results and Discussion

**Figure 1:**
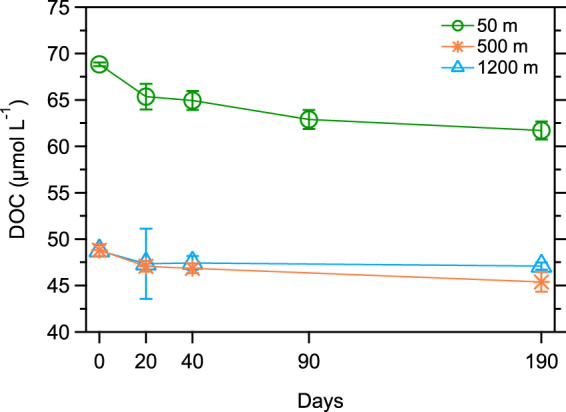
Microbial utilization of DOC in surface (50 m), mesopelagic (500 m), and deep (1200 m) waters of the Atlantic Ocean. The x-axis shows the duration of incubation on a linear scale. Data are reported as the average value and standard deviation (*n* = 3).

Bioassay experiments with seawater collected from three depths in the Atlantic Ocean revealed a wide range of DOC reactivity (Fig. 1). About 10% of the DOC in surface water was utilized by microbes over six months, whereas only ~3% of the DOC in deep water was removed. There was a relatively rapid removal of DOC during the first 20 days, indicating the occurrence of labile components of DOC at all depths. The labile fractions of DOC accounted for about half of the total DOC removal in surface and mesopelagic waters, whereas nearly all the DOC removal in deep water occurred within 20 days. This observation is consistent with a small fraction of ^14^C-enriched (i.e. modern) and potentially labile DOC in the deep Atlantic Ocean^18^. Sinking particles formed in productive surface waters are likely sources of labile DOC in mesopelagic and deep waters where solubilization processes release bioavailable dissolved organic matter (DOM)^10,19^. The bulk (≥90%) of the DOC throughout the water column remained at the end of the incubations, confirming the current view that the majority of natural DOC is very resistant to microbial degradation.

Solid phase extraction (C-18) preferentially isolates nonpolar compounds commonly found in marine humic substances, such as carboxylated aliphatic molecules^20^. Carboxyl-rich alicyclic molecules (CRAM) are a diverse mixture of carboxylated molecules that comprise about 8% of the DOC in seawater^11^. The CRAM components of DOC appear to be old and refractory components of the deep ocean DOC reservoir^12,21^. The C-18 extraction process recovered 29–30% of the DOC from surface, mesopelagic and deep waters. The average C/N of the isolated DOM (36.6 ± 1.3) was similar to that of humic substances extracted from seawater using XAD-2 resin^20^ and is indicative of CRAM and other N-depleted aliphatic molecules.

**Figure 2:**
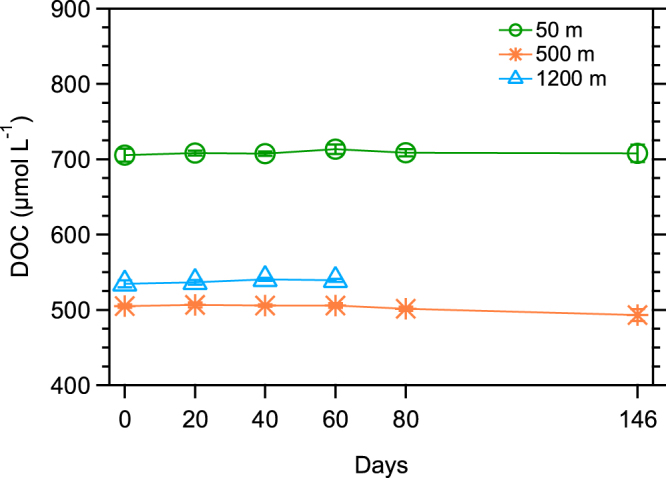
Microbial utilization of C-18 DOC in surface (50 m), mesopelagic (500 m), and deep (1200 m) waters of the Atlantic Ocean. DOC was isolated using C-18 solid phase extraction, and the C-18 DOC was added to the incubations at high concentrations (~10-fold higher than *in situ* values). Microbial inocula were collected from the same depths as the C-18 DOC and added (1:50) to the incubations. The x-axis shows the duration of incubation on a linear scale. Data are reported as the average value and standard deviation (*n* = 3).

The bioreactivity of the C-18 extracted DOC was examined using bioassay experiments with artificial seawater amended with nitrate, phosphate and microbial assemblages collected from the corresponding depths. The extracted DOC was added to incubations as the sole carbon and energy source at high concentrations (500–700 µM C), which were ~10-fold greater than *in situ* concentrations. The concentrations of the isolated DOC showed minor changes throughout the incubations at all depths (Fig. 2), in contrast with the experiments showing microbial utilization of more reactive components of bulk DOC in waters from the same depths (Fig. 1). Microbes would utilize elevated concentrations of labile molecules in the C-18 DOC if any were present, so it appears intrinsic properties of molecules limited microbial utilization. Results from the bioassay experiments are consistent with the refractory nature of CRAM^11,12^ and the characterization of C-18 isolated DOC as refractory DOC.

The observations of the refractory nature of C-18 extracted DOC are in contrast with those of a recent study^13^ examining the microbial utilization of high concentrations of DOC extracted from deep waters using a modified styrene-divinylbenzene polymer (PPL), which isolates more polar molecules than C-18. Microbial growth was observed to increase with increasing concentrations (up to 10-fold) of PPL extracted DOC^13^, thereby providing evidence that the concentrations of some components of DOC were below threshold concentrations required for microbial utilization and growth^14^. The PPL extraction used by Arrieta *et al*.^13^ recovers a greater fraction of seawater DOC (≥40%) than the C-18 extraction (≤30%) and includes more N-containing molecules compared to the C-18 extraction^22^. As demonstrated in the present bioassay experiments with unamended seawater, ~3% of the DOC in deep water was labile and rapidly utilized by microbes (Fig. 1). It is possible the PPL extraction concentrates some of these labile components of deep water DOM, thereby supporting the growth of microbes observed during short-term (10–20 days) bioassay experiments. A recent modeling analysis of the radiocarbon signature of DOC suggests low concentrations of labile molecules likely account for a relatively small fraction of the DOC reservoir^23^. Our observations of the refractory nature of C-18 isolated DOC are consistent with these modeling results.

**Figure 3:**
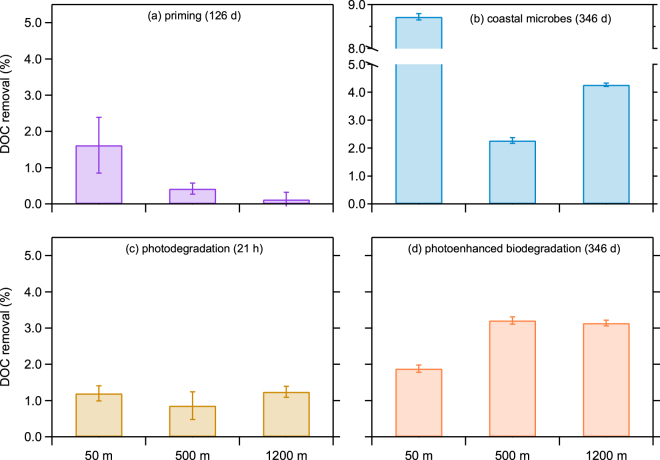
Removal of C-18 DOC under different conditions. (**a**) The effect of priming by a labile carbon and energy source (glucose). (**b**) Biodegradation by a coastal microbial assemblage. (**c**) Photodegradation during 21 h of irradiation in a solar simulator. (**d**) Microbial utilization of DOC following the irradiation, corrected for dark controls. Data are reported as the average value and standard deviation (*n* = 3).

Microbial degradation of refractory organic matter can be limited by the availability of substrates that support metabolism and growth^24,25^. Therefore, a labile carbon and energy source (glucose) was added to replicate experiments to investigate the priming effect on refractory DOC removal. Glucose utilization occurred during the initial stage of the experiments (Supplementary Table 1), indicating labile DOC was readily utilized by an active microbial community. Glucose amendments resulted in a net removal of 0.1–1.6% of the refractory DOC after 126 days of incubation (Fig. 3a). The addition of a labile substrate stimulates microbial metabolism and enzyme production thereby facilitating the degradation of refractory molecules^25^. These experiments demonstrate a priming effect on the biodegradation of refractory DOC and are consistent with a previous study showing enhanced utilization of DOC when surface waters from the Sargasso Sea were amended with glucose, ammonia and phosphorous^26^.

Microbial community structure and metabolic diversity vary across ocean biomes and throughout the water column^27–29^. This spatial heterogeneity in metabolic diversity influences the types of molecules on the menu for microbial consumption in different water masses. The accumulation of DOC occurs in surface waters of the Sargasso Sea during the productive spring and summer months, and this surplus of DOC is utilized by microbial communities in upper mesopelagic waters following winter convection^30,31^. Given these observations, we replaced the native microbial assemblage in the bioassay experiments with an assemblage collected from a coastal area to investigate their utilization of refractory DOC. Coastal seawater includes a diverse assortment of versatile microbes that can metabolize a wide range of substrates^32^. Coastal microbes consumed 2.3–8.7% of the refractory DOC at all depths after 346 days of incubation (Fig. 3b), demonstrating varying metabolic capabilities among microbial communities from different habitats.

Solar radiation drives photochemical reactions in surface waters that degrade and alter organic molecules and enhance microbial utilization and growth^33,34^. Refractory DOC in the bioassay experiments was irradiated for 21 h in a solar simulator to investigate photomineralization and the production of bioavailable photoproducts. Following irradiation, chromophoric components of the refractory DOC showed a major reduction (≥55%) in ultraviolet light absorption (*a*_350_) and an increase (>50%) in the spectral slope coefficient (*S*_275-295_) (Supplementary Fig. 2), indicating substantial photobleaching and the production of low-molecular-weight photoproducts. Photodegradation directly mineralized 0.9–1.2% of the refractory DOC from each depth (Fig. 3c), and microbial utilization of bioavailable photoproducts removed an additional 1.9–3.2% of the refractory DOC after 346 days of incubation (Fig. 3d). The microbial utilization of photoproducts was rapid during the first week following irradiation and demonstrated the conversion of refractory molecules into labile forms (Supplementary Fig. 3). Rates of DOC utilization in the irradiated and dark controls declined over time, indicating a broad spectrum of molecular reactivity within the refractory DOC pool.

**Figure 4:**
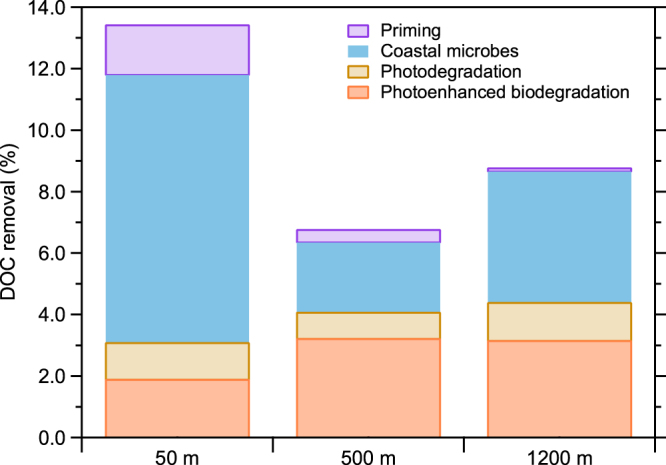
Cumulative removal of C-18 DOC under various environmental conditions and with different microbial communities.

Overall, a surprisingly large fraction of refractory DOC was removed during experiments of relatively short duration. The combined experimental treatments resulted in the removal of 7–9% of refractory DOC from deep and mesopelagic waters and 13% from surface waters (Fig. 4). It is interesting to note that photodegradation and photoenhanced biodegradation, which only occur in the surface ocean, were responsible for over half of the total removal of DOC from deeper waters (500 m and 1200 m). Bacterial utilization of old DOC has been observed in the surface ocean^35^, and it appears this process is mediated by the photodegradation of refractory DOC^36^. These observations indicate global overturning circulation plays an important role in the removal of deep-sea DOC.

**Figure 5:**
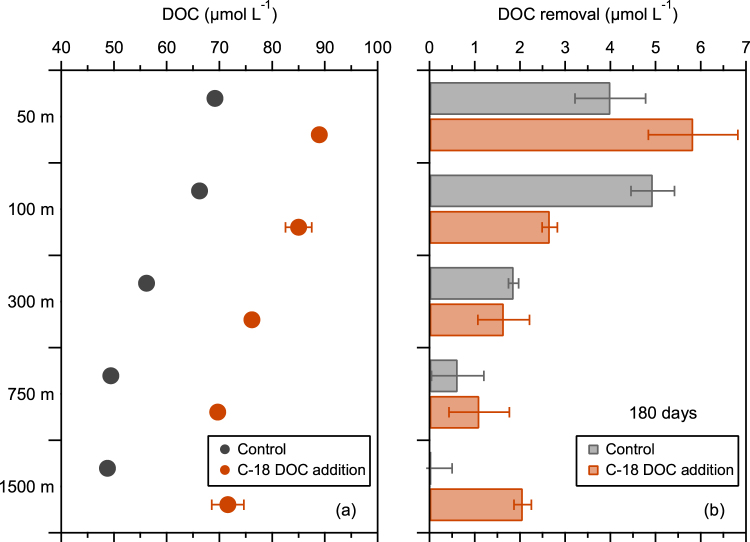
(**a**) Depth profile of DOC concentrations at BATS. Seawater samples were collected from 50, 100, 300, 750, and 1500 m and were divided into two treatments: unamended (control) and amended with ~20 µmol L^−1^ of C-18 DOC isolated from 1200 m (as in Fig. 2). (**b**) Removal of DOC in the control and amended groups after 180 days of dark incubation at room temperature (22–24 °C). Data are reported as the average value and standard deviation (*n* = 3).

The removal of refractory DOC was further investigated using a different experimental design. The C-18 isolated DOC from deep water (1200 m) was added (~20 µmol L^−1^) to unfiltered seawater collected from five depths (50–1500 m) at the Bermuda Atlantic Time-Series Study (BATS) site (Fig. 5a) and incubated with 3 replicates in the dark for 180 days with unamended controls (Fig. 5b). Microbial communities and nutrient availability vary with depth at the BATS site^37,38^, thereby providing exposure of C-18 DOC to diverse microbes and environmental conditions. Comparisons in the total DOC removal between the control and amendment showed negligible net changes (<0.5 µmol L^−1^) in concentrations of C-18 DOC at three of the five depths (100, 300, and 750 m; Fig. 5b). This confirms the refractory nature of the C-18 DOC. A small but significant removal of C-18 DOC was observed at 50 m and 1500 m (by ~2 µmol L^−1^; Mann–Whitney *U*-test, *p* < 0.05), indicating potential removal of refractory DOC when exposed to different microbes and environmental conditions.

**Figure 6:**
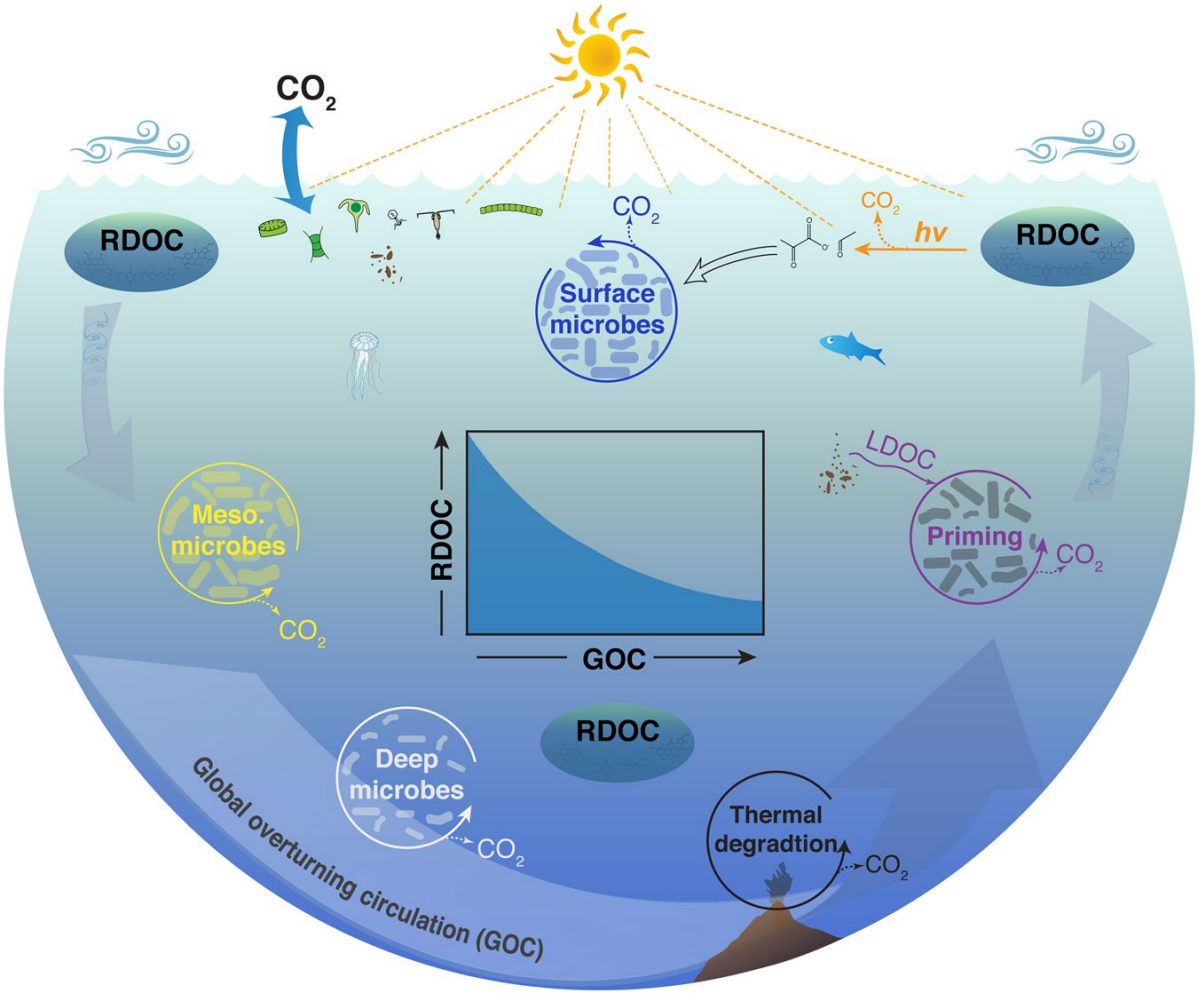
Conceptual diagram illustrating major processes regulating the removal of refractory DOC in the ocean. Phytoplankton production and food web dynamics in surface waters release a diverse mixture of dissolved molecules with varying reactivities. Bacteria and archaea utilize labile and semi-labile forms of DOC in surface and mesopelagic (Meso.) waters of the upper ocean, leaving behind a vast reservoir of refractory DOC (RDOC) that persists in the ocean for millennia. The ocean is a patchy environment that harbors a great diversity of microbes and physicochemical processes with the potential to remove refractory DOC when these molecules encounter environmental conditions and microbes that can degrade them. Physical mixing transports refractory DOC throughout the ocean realm and thereby increases the likelihood of its removal. Deep ocean waters can be entrained into hydrothermal circulation and associated DOC can be removed by thermal degradation. Sinking particles from the upper ocean release labile DOC (LDOC) that triggers hot spots of microbial activity and primes the removal of refractory molecules. Mixing of subsurface waters into sunlit waters exposes refractory DOC to warmer temperatures and photochemical processes that can mineralize and transform refractory molecules into simple compounds (e.g., pyruvate, formaldehyde) for rapid microbial utilization. Thus, it appears the lifetime of refractory molecules in the ocean is regulated by the rate of global overturning circulation (GOC). This relationship indicates a slowing of GOC could lead to an increase in the reservoir size of refractory DOC, assuming a constant production rate of refractory DOC (inset panel). Symbols used in the figure are obtained or modified from U.S. JGOFS, Buesseler *et al*.^57^, Vecteezy.com, and courtesy of the Integration and Application Network, University of Maryland Center for Environmental Science (ian.umces.edu/symbols/).

Rapid experimental manipulations introduced different microbial communities and environmental conditions that would normally occur in the ocean over much longer periods of time through mixing processes and large-scale overturning circulation. The ocean is a spatially and temporally patchy environment, with microscale processes dominating DOC transformations^10,39^. Refractory molecules persist in the ocean, yet when they encounter their fate removal is relatively swift. Photosynthesis, grazing, viral lysis and the decomposition of sinking particles, release microscale plumes of labile substrates that can serve as priming agents for microbial degradation of refractory molecules (Fig. 6). These events generate hot spots and hot moments of microbial interactions, elevated rates of microbial activity, and the potential removal of refractory molecules. The patchiness of microscale habitats in the ocean enhances microbial diversity^39^ and likely expands the metabolic potential for the removal of refractory molecules. There is a growing awareness of the vast diversity and metabolic potential in the rare biosphere^29,40^, a world of sparsely-distributed, slow-growing microbes that could play an important role in the degradation of refractory molecules.

Marine heterotrophic microbes have dual roles as consumers and producers of DOC in the ocean carbon cycle. Most of the DOC they consume is respired to carbon dioxide, but a small fraction is transformed into refractory DOC that sequesters carbon in the ocean for ages, a process known as the microbial carbon pump^41,42^. In this process, bacteria, and presumably archaea, produce a potpourri of diverse molecules, such as CRAM, which are released into the water column primarily through cellular exudation, grazing and viral lysis^43–45^. Decomposition processes alter the composition and decrease the size of organic matter, thereby shaping the size-composition-age-reactivity continuum of the DOM reservoir^4,46,47^. Further utilization of these molecules may require a suite of favorable environmental conditions (e.g., priming substrates, sunlight) and the collective metabolic activities of various microbial specialists^48^.

A bewildering diversity of microorganisms, microhabitats and dissolved organic molecules coexist in the ocean realm. Although separated in space and time, ocean mixing at various scales can transport refractory DOC great distances, much further than the molecules or microbes could explore by diffusion or chemotaxis^39^. Mixing exposes refractory molecules to varying environmental conditions and microbial communities, and it facilitates their degradation and removal (Fig. 6). Molecules swept along the seabed can be entrained into hydrothermal vent systems and pyrolyzed^16,17^. Upwelling delivers DOC to sunlit and warmer surface waters where photochemical and microbial processes transform and mineralize organic molecules^34,36^. Refractory molecules that escape the gauntlet of removal processes during one pass through global ocean circulation enter the next round, and on average seem to survive several mixing cycles. Thus, it seems the lifetime of refractory molecules is regulated by the rate of global overturning circulation, with a faster circulation leading to a faster turnover of refractory DOC in the ocean.

Global overturning circulation appears to play a key role in regulating the size of the ocean reservoir of refractory DOC (Fig. 6 inset). Geological records of carbon isotopic variability are thought to record major fluctuations in the ancient ocean DOC reservoir induced by periodic changes in ocean circulation, stratification, and periods of anoxia^49,50^. The warming and freshening of surface waters in the modern ocean over the past several decades is causing a slowdown of overturning circulation^51^, and this trend is projected to continue for a few millennia^52^. The potential influence of climate change on the efficiency of the microbial carbon pump appears to be multifaceted, and the net effect on the production of refractory DOC is unclear^42,53^. Assuming a constant production rate, the ocean reservoir of refractory DOC could increase during a weakening of global overturning circulation, providing a negative feedback to rising concentrations of atmospheric carbon dioxide. Recognition of the interconnections between the ocean reservoir of DOC and overturning circulation provides further insight about potential impacts of climate change on the ocean carbon cycle.

## Methods

### Sample collection and preparation

Sampling was conducted in the North Atlantic Ocean (35°39.847N, 74°30.792W) on the R/V *Neil Armstrong* in March 2016. The upper water column in this region is influenced by the northeastward flowing Gulf Stream, and the southward flowing North Atlantic Deep Water dominates the water column below 1000 m. Water samples were collected from surface (50 m), mesopelagic (500 m), and deep waters (1200 m) using Niskin bottles mounted on a CTD rosette sampler. At each depth, 80 L of seawater was collected in acid-cleaned high-density polyethylene carboys for solid-phase extraction, 250 ml of seawater was collected in clean (400 °C for 4 h) Kimax glass bottles (3 replicates per depth) for microbial degradation experiments, and 120 ml of seawater was collected in clean glass vials for a microbial inoculum.

### Solid-phase extraction of DOC

Water samples for solid-phase extraction were filtered onboard through 0.2 µm Nuclepore polycarbonate cartridge filters (pre-rinsed with Milli Q water and seawater) and acidified to pH 2.5 with 6 mol L^−1^ sulfuric acid. Acidified samples from each depth were passed through C-18 cartridges (Agilent Bond Elut; 10 g) at a flow rate of 50 ml min^−1^ and 20 L per C-18 cartridge. Cartridges were pre-conditioned with methanol (HPLC grade) and Milli Q water (pH 2.5) immediately prior to use. The extracted DOC was eluted from each cartridge in 30 ml of methanol and dried under a gentle stream of N_2_. The C-18 extraction recovered 29 ± 1% of the bulk DOC and 53 ± 6% of the CDOM absorption coefficient at 350 nm (*a*_350_).

### Bulk and refractory DOC degradation experiments

Microbial utilization of bulk DOC was determined using bioassay experiments with unfiltered seawater. Water samples (~250 ml) were incubated in glass bottles at room temperature (21–24 °C) in the dark immediately after collection. Subsamples for DOC analysis were taken on days 0, 20, 40, 90, and 190. All experiments in this study were conducted with 3 replicates.

Biological and photochemical degradation of refractory DOC were determined in a series of laboratory experiments (Supplementary Fig. 1) using C-18 extracted DOC (i.e. refractory DOC) and carbon-free artificial seawater (ASW). Preparation of ASW followed the procedure described by Lechtenfeld, *et al*.^43^. Six replicate clean Kimax glass bottles, each containing 300 ml of ASW, were used for each depth. Dried C-18 DOC from 50 m, 500 m, and 1200 m was re-dissolved in ASW, passed through a glass fiber filter (GF/F, 0.7 µm pore-size; 400 °C for 4 h), and mixed into the bottles at final concentrations of 707, 508, and 535 µmol C L^−1^, respectively. Inorganic nutrients (NaNO_3_ and KH_2_PO_4_) were added at final concentrations of 48.5 µmol N L^−1^ and 3.0 µmol P L^−1^. Natural microbial inocula collected from these depths were added to the corresponding bottles at a 1:50 (v/v) dilution within a week of collection.

The six replicates at each depth were divided into two groups: a control group and a priming group amended with ~120 µmol C L^−1^ of glucose on day 20 (Supplementary Table 1). Subsamples for DOC analysis were collected on days 0, 20, 40, 60, 80, and 146. On day 61, a fresh microbial inoculum was added to the deep-water experiments to investigate if microbes from a different location and depth could degrade the C-18 DOC (Supplementary Table 1). The priming effect (Fig. 3a) was calculated as the difference in total DOC removal (%) between the control (*C*) and priming groups (*P*) as in Eq. (1):

1 $$Priming\,({\rm{ \% }})={[\frac{DO{C}_{0d}-DO{C}_{146d}}{DO{C}_{0d}}\times 100]}_{P}-\,{[\frac{DO{C}_{0d}-DO{C}_{146d}}{DO{C}_{0d}}\times 100]}_{C}.$$

where DOC_0d_ and DOC_146d_ are concentrations of DOC on day 0 and day 146. It has been noted that a small fraction (~5%) of glucose is transformed into refractory DOC during microbial utilization and production^41^. Therefore, the net change (%) calculated using Eq. (1) likely underestimates the true priming effect by 0.9%, 1.2% and 0.5% for the surface, mesopelagic and deep-water experiments, respectively.

The experiments were ended after 146 days by passing the samples through Supor membranes (0.2 µm pore-size) to remove microbial populations. Filtered seawater from the priming group received a freshly collected coastal microbial assemblage (0.2–1.5 µm; added at a 1:20 dilution; same below), and was incubated in the dark at room temperature (22–24 °C) for another 346 days (Supplementary Fig. 3). The removal of DOC by coastal microbes (Fig. 3b) was calculated as the percentage difference in DOC concentrations between day 0 and day 346, as in Eq. (2):

2 $$DOC\,removal\,by\,coastal\,microbes\,( \% )=\frac{DO{C}_{0d}\,-\,DO{C}_{346d}}{DO{C}_{0d}}\times 100.$$

Filtered seawater from the control group was transferred into clean quartz flasks for irradiation (photodegradation). The irradiation was conducted in an Atlas Suntest XPS+ solar simulator at 750 W m^−2^ for 21 h, a duration equivalent to ~2 months of natural solar exposure in the surface mixed layer of the ocean^54^. The concentration of DOC and CDOM absorbance were measured before and after the irradiation (Supplementary Fig. 2). The removal of DOC by photodegradation (Fig. 3c) was calculated as the percentage difference in DOC concentrations between time zero and time final (21 h), as in Eq. (3):

3 $$Photodegradation\,( \% )=\frac{DO{C}_{0}\,-\,DO{C}_{21h}}{DO{C}_{0}}\times 100.$$

The coastal microbial assemblage was then added to the irradiated group and the dark control group, and both were incubated in the dark at room temperature (22–24 °C) for 346 days (Supplementary Fig. 3). The photoenhanced microbial utilization of DOC (Fig. 3d) was calculated by subtracting the total DOC removal (%) in the irradiated groups (*I*) from values in the dark controls (*C*; same values as those calculated from Eq. 2), as in Eq. (4):

4 $$Photoenhanced\,biodegradation\,( \% )={[\frac{DO{C}_{0d}-DO{C}_{346d}}{DO{C}_{0d}}\times 100]}_{I}-{[\frac{DO{C}_{0d}-DO{C}_{346d}}{DO{C}_{0d}}\times 100]}_{C}.$$

### Chemical and optical analyses

Samples for DOC and CDOM analyses were filtered through GF/F filters (400 °C for 4 h) immediately after collection. Concentrations of DOC were measured by high temperature combustion using a Shimadzu total organic carbon TOC-V analyzer equipped with an autosampler^55^. Blanks (Milli Q Plus water) were negligible throughout the measurements^56^. Deep-sea reference standards were injected every 6th sample to monitor the accuracy and consistency of the analysis. The coefficient of variation among injections of a given sample was typically within ±1%.

Absorbance spectra (250–800 nm) were measured using a Shimadzu ultraviolet-visible 1601 dual beam spectrophotometer and 1-cm path length quartz cuvettes. The absorbance (*A*_λ_) at a wavelength of λ was blank corrected (minus the average absorbance between 690–700 nm) and converted to a Napierian absorption coefficient, *a*_λ_ (m^−1^): *a*_λ_ = 2.303 *A*_λ_/0.01. The spectral slope coefficient (*S*) was calculated as the slope of the linear regression of log-linearized *a*_λ_: *a*_λ1_ = *a*_λ2_ exp [−*S* (λ_1_ − λ_2_)], where *a*_λ1_ and *a*_λ2_ are absorption coefficients at wavelengths of λ_1_ and λ_2_. The spectral slope coefficient between 275 and 295 nm is reported as *S*_275–295_ with a unit of µm^−1^.

### Data availability

All data generated or analyzed during this study are included in this published article (and its Supplementary Information files).

## Electronic supplementary material

Supplementary material
