# Supplementary material for: Mixing it up in the ocean carbon cycle and the removal of refractory dissolved organic carbon
Source: Sci Rep. 2018 Feb 7;8:2542. doi: 10.1038/s41598-018-20857-5 (PMC5803198; doi:10.1038/s41598-018-20857-5)
Supplement: Supplementary file 1 — Supplementary material [file 41598_2018_20857_MOESM1_ESM.pdf]

Mixing it up in the ocean carbon cycle and the removal of refractory dissolved organic carbon

Yuan Shen[1†], and Ronald Benner[1,2*]

[1]School of the Earth, Ocean and Environment and [2]Department of Biological Sciences, University of South Carolina, Columbia, South Carolina 29208

[†]Present address: Ocean Sciences Department, University of California, Santa Cruz, California 95064

[*]Corresponding author: Ronald Benner (benner@mailbox.sc.edu)

Supplementary Table 1. Concentrations of C-18 extracted dissolved organic carbon (DOC) in the control and priming experiments.

Depth	Treatment	Day 0	Day 20	Day 20*	Day 40	Day 60	Day 61†	Day 80	Day 146
50 m	Control	705±8	708±3	-	707±3	713±6	-	709±5	708±11
50 m	Priming	708±4	707±10	828±10	772±49	713±4	-	713±6	697±4
500 m	Control	505±2	507±3	-	506±2	506±3	-	501±3	493±8
500 m	Priming	511±3	512±3	633±4	581±62	538±53	-	503±3	496±2
1200 m	Control	535±5	536±3	-	541±2	539±2	329±3	333±3	324±1
1200 m	Priming	543±3	541±2	662±1	660±1	656±1	400±5	340±4	329±1

*Glucose (~120 µmol L^{-1}) was added to the priming group immediately following the subsampling on day 20. †On day 61, a fresh microbial inoculum was added to the deep-water experiments to investigate if an inoculum from a different location and depth could degrade the C-18 extracted DOC. Unfiltered seawater was collected from surface (50 m) and mesopelagic (400 m) waters at the Bermuda Atlantic Time-Series Study (BATS) site. The surface and mesopelagic waters were combined (1:1; v/v) and added (10:13 dilution) to the deep-water control and priming group incubations within a week of collection. This addition introduced a diverse mixture of microbes and nutrients to the experiments and resulted in ~1% net removal of DOC in the controls between days 61-146, confirming the refractory nature of C-18 DOC. Data are reported as the average value and standard deviation (n = 3).

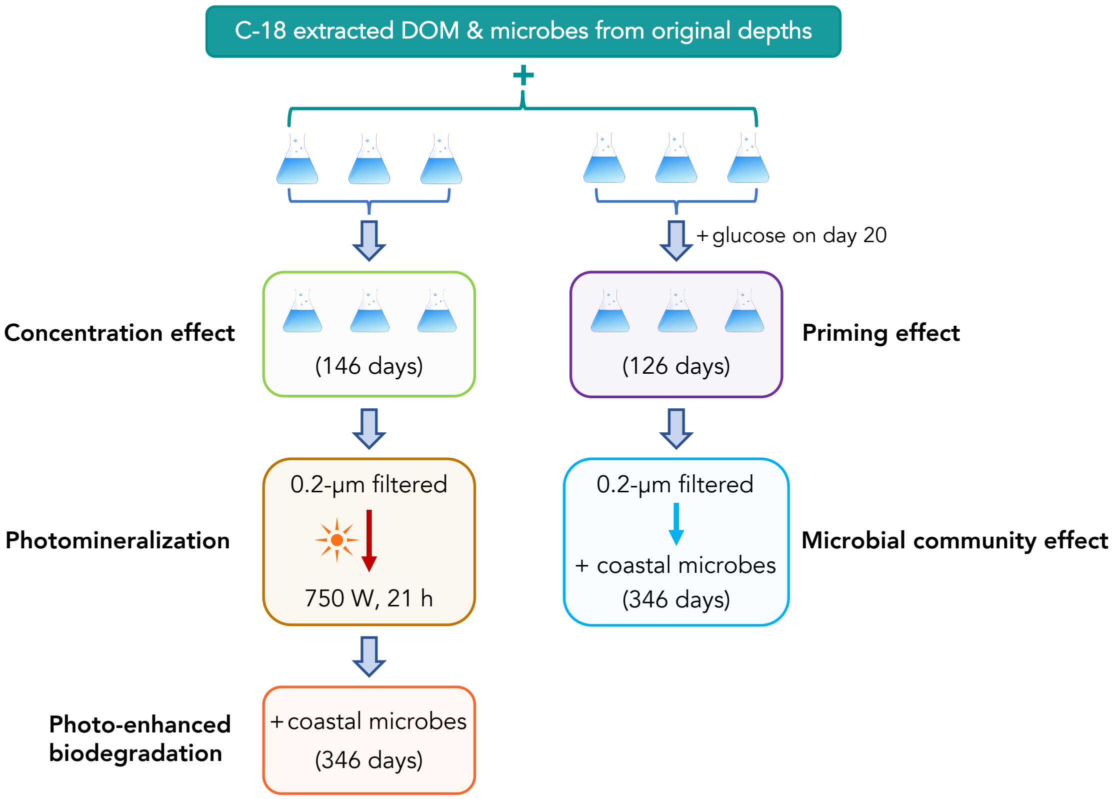

Supplementary Figure 1. Flow chart of the experiments designed to determine the removal of refractory DOC under varying environmental conditions and with different microbial communities. Details of each experiment are provided in the Methods. All experiments are conducted with three replicates.

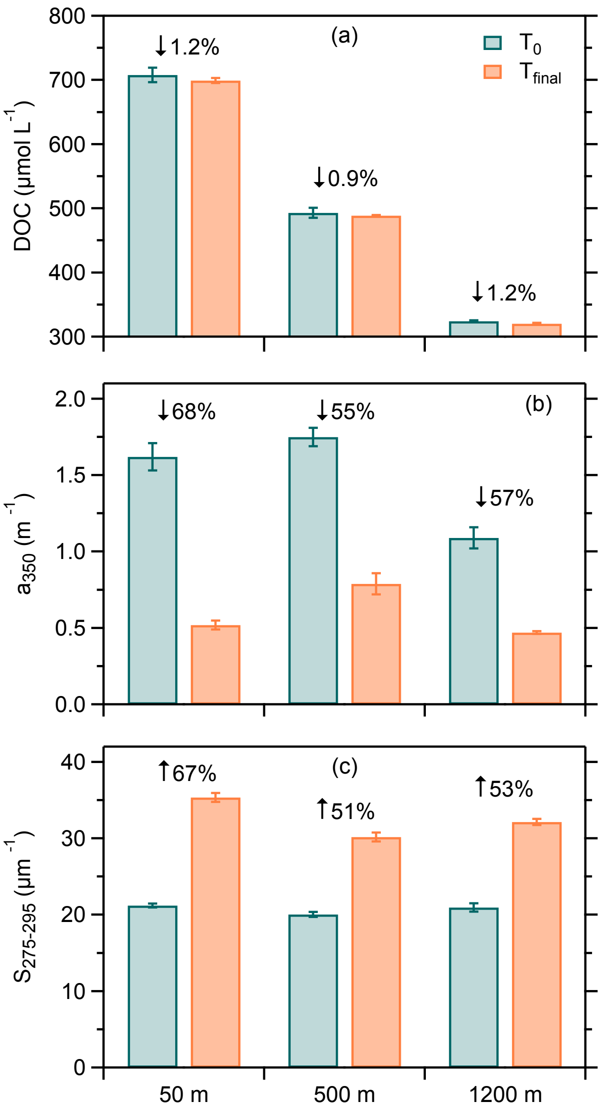

Supplementary Figure 2. Changes in concentrations and chromophoric properties of C-18 extracted dissolved organic carbon (DOC) after 21 h of irradiation in a solar simulator. (**a**) concentrations of DOC. (**b**) chromophoric DOM absorption coefficient at 350 nm (a_{350}). (**c**) spectral slope coefficient between 275 and 295 nm ($S_{275\text{-}295}$). The average percentage change is labeled for each sample set (n = 3). Error bars represent the standard deviation. Note that the unit of $S_{275\text{-}295}$ is converted from nm^{-1} to μm^{-1} by multiplying by one thousand.

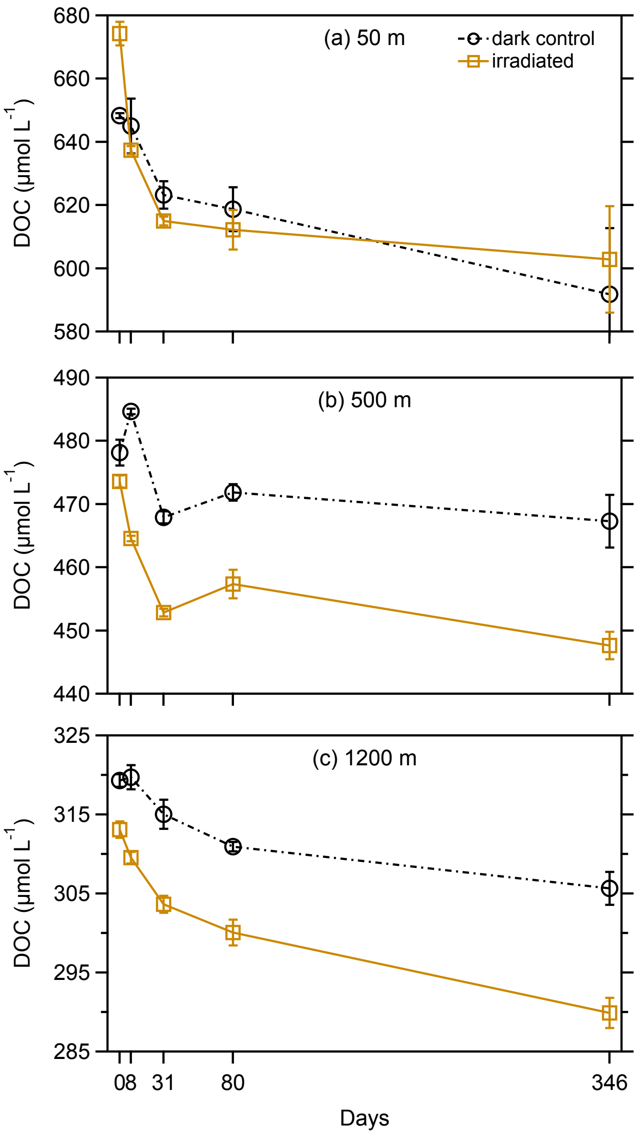

Supplementary Figure 3. Changes in concentrations of C-18 extracted dissolved organic carbon (DOC) during 346 days of incubations in the dark control and irradiated groups. Both experiments were initiated by the addition of the same coastal microbial assemblage (see the Methods). Data are reported as the average value and standard deviation (*n* = 3).
